# Impact of Quantitative Videofluoroscopic Swallowing Measures on Clinical Interpretation and Recommendations by Speech-Language Pathologists

**DOI:** 10.1007/s00455-023-10580-3

**Published:** 2023-05-01

**Authors:** Gwen Kerrison, Anna Miles, Jacqui Allen, Michael Heron

**Affiliations:** 1 The University of Auckland - Waipapa Taumata Rau, 22 Symonds Street, Auckland, 1010 New Zealand; 2 Te Whatu Ora - Hauora a Toi Bay of Plenty, 829 Cameron Road, Tauranga, 3112 New Zealand; 3 Te Whatu Ora - Waikato, Pembroke Street, Hamilton, 3204 New Zealand; 4 School of Psychology, The University of Auckland- Waipapa Taumata Rau, Grafton Campus, Park Road, Private Bag 92019, Auckland, New Zealand; 5 Auckland ENT Group, 242 Great South Road, Greenlane, Auckland, New Zealand; 6 Te Whatu Ora - Te Matau a Māui Hawkes Bay, Corner Omahu Road and McLeod Street, Private Bag 9014, Hastings, 4156 New Zealand

**Keywords:** Dysphagia, Deglutition, VFSS, MBS, Quantitative analysis, Reliability, Rehabilitation

## Abstract

Quantitative measures are available for adult videofluoroscopic swallow study (VFSS) analysis but are yet to be seen routinely in clinical practice. This study explores agreement between traditional observational analysis and quantitative analysis, and the impact of analytical approaches on subsequent diagnosis and recommendations. One hundred adults referred for VFSSs with swallowing concerns were administered a standardised VFSS protocol. All VFSSs were analysed using three approaches: (1) a traditional observational analysis typically used by treating speech-language pathologists (SLPs), (2) quantitative analysis by two independent raters, and (3) binary subjective analysis by 11 independent raters. Three metrics were focussed on; pharyngeal constriction (PC), hyoid displacement (Hmax) and pharyngoesophageal segment opening (PESmax). All raters were blinded to others’ ratings. Treating SLPs using traditional observational analysis were provided with no instructions. Quantitative analysis used published Leonard and Kendall digital displacement measures. Binary subjective analysis involved rating each VFSS as normal versus impaired for the three metrics above. Treating SLPs using traditional observational analysis and quantitative analysis raters independently provided diagnostics and treatment plans. PC, Hmax and PESmax achieved fair agreement (Kappa = 0.33–0.36) between binary subjective analysis compared to substantial agreement (ICC = 0.77–0.94) for quantitative analysis. Reports of impairment were significantly lower in the traditional observational and binary subjective analyses compared with studies rated using the quantitative analysis (*p* < 0.05). Consequently, this resulted in significantly less rehabilitation recommendations when traditional observational analysis was used in comparison to the quantitative analysis. Quantitative measures to analyse VFSSs can be used in clinical practice producing increased inter-rater agreement and supporting more targeted rehabilitation recommendations than using a traditional observational VFSS analysis alone.

## Introduction

Videofluoroscopic Swallow Study (VFSS) is a widely available instrumental swallowing assessment used by speech-language pathologists (SLPs) [1, 2]. Instrumental assessment is required to analyse swallowing biomechanics in order to provide evidence-based, tailored management plans for patients [1–4]. VFSS allows in-depth dynamic assessment of biomechanics, swallowing function and airway protection with the ability to trial compensatory management techniques and guide rehabilitation. However, for decades, VFSS has been criticised for poor objectivity and inter-rater agreement [5–7].

There are many likely causes of this poor inter-rater agreement, including insufficient frame rate, poor quality of equipment and recording, inability to play the video frame-by-frame, education, training and rater experience [5–7]. In the past three decades, there have been attempts to improve reliability by applying standardisation to how VFSSs are conducted and reported [2, 4, 8–11].

### Subjective Observational Approach

Traditionally, VFSS interpretation relied on subjective, observational analysis by an SLP [1, 2]. The development of criterion-referenced analysis tools such as the penetration-aspiration scale (PAS) [8] and the Modified Barium Swallow Impairment Profile (MBSImP) [4] have led to improved agreement across trained SLPs. More recently, Dynamic Imaging Grade of Swallowing Toxicity (DIGEST) has been developed which combines grading of swallow safety and efficiency to provide an overall grade of severity [9]. While these move towards consistency in definitions to aid interpretation and greater inter-rater agreement, analysis continues to depend on an element of observational skill and subjectivity.

### Quantitative Measures

Standardised quantitative VFSS protocols such as Dynamic Swallow Study (DSS) developed by Leonard and Kendall [2] and Analysis of Swallowing Physiology: Events, Kinematics & Timing (ASPEKT) reported by Steele et al. [10] have also been developed. These allow quantification ideal for repeated measures across patients and time. Within the last 25 years, these quantitative approaches have been explored in both healthy and dysphagic populations providing normative data for comparison with patient populations and established strong inter-rater reliability assurance [2, 3, 10, 12–18]. Quantitative measures also provide the ability to predict the chance of aspiration or development of aspiration pneumonia even if aspiration is not observed during the study [2, 10, 19–21]. These timing, distance and area measures can be made through simple additions to the VFSS protocol, such as a millisecond timer interfaced onto the recorder, frame-by-frame analysis and a calibration ring (a metal ring of known diameter enabling calibration of spatial measures after the study) [2, 10] or scaling of measurements to the length of the patient’s C2-5 cervical spine [2, 3, 10]. These quantitative measures are obtained by direct measurement of landmarks on VFSS frames, translating to physical lengths or areas, or in the case of timings, durations in milliseconds.

### Reliability of VFSS Interpretation

Where quantitative measures have been used as a gold standard, subjective observational analysis performs poorly in comparison. In one study, percentage accuracy varied from 19 to 76% across various physiological elements [21]. Similarly, when SLPs and laryngologists were asked to rate 76 VFSSs as normal or impaired for hyoid elevation (HE), pharyngeal area (PA), pharyngeal constriction ratio (PCR) and pharyngoesophageal segment maximum opening (PESmax), raters correctly classified only 62% of VFSSs, with only moderate inter-rater agreement [22]. An early study by Nordin et al., explored competency development in SLPs learning quantitative measures. After 8 weeks of measuring three videos per week, all SLPs achieved 80% accuracy and took only 20 min to complete a VFSS report per patient. Inter-rater agreement was substantial (ICC range 0.71–0.98) [12]. Yet, despite the demonstrated acceptable timeframe to complete VFSS analysis using quantitative analysis, anecdotally, this method does not seem to be commonly used in clinical practice [11]. While lack of time, resources, training and confidence in using quantitative methods may be barriers to wider implementation, another prevailing belief is that SLPs can garner all needed information from more traditional observational approaches i.e. watching the study in the radiology suite or in the clinic afterwards and writing a summarised subjective report [12].

### Proposed Study

There is minimal data published to demonstrate the impact of quantitative measurement in VFSS on clinician agreement, diagnostic accuracy, management decisions and patient outcomes [10, 20]. In this study, agreement between standard practice subjective, observational VFSS analysis and quantitative analysis was explored. This study sought to explore the following questions: does the introduction of quantitative measures into VFSS analysis change the rate and type of physiological impairments identified by clinicians and, does the introduction of quantitative measures into VFSS analysis change recommendations made by clinicians? Three metrics were focused on pharyngeal constriction (PC), hyoid displacement (Hmax) and pharyngoesophageal segment opening (PESmax). Our hypotheses were that there would be discrepancies in diagnoses and rehabilitative recommendations when patients are assessed using traditional subjective analysis compared to quantitative VFSS measurement and there would be substantial intra- and inter-rater agreement when clinicians use quantitative VFSS measurement and poorer inter-rater agreement for those using observational analysis.

## Methods

This project gained national ethics approval from The University of Auckland Human Participants Ethics Committee (UAHPEC 9263).

### VFSS Samples

A sample of 140 consecutive VFSSs performed on patients referred to SLP at one regional hospital for investigation of swallowing problems between August 2015 and September 2016 were collected. Forty videos were excluded, either due to inadequate imaging quality to enable quantitative measures to be completed (*n* = 30) or the patient had already participated in the study (*n* = 10) bringing the total VFSSs for inclusion to 100 (60 males; mean age 72 years, standard deviation (SD) 13.7, range 34–93 years). Patient diagnosis, past medical history and demographics were collected from the hospital clinical database. Patients were broadly classified by the most likely medical diagnosis contributing to their swallowing issues: neurological (*n* = 29), nil known diagnosis (*n* = 21), gastroenterology complaint (*n* = 13), respiratory complaint (*n* = 10), head and neck cancer (*n* = 8), otorhinolaryngology complaint (*n* = 8), cardiology complaint (*n* = 5) and other e.g. tracheostomy and inclusion body myositis (*n* = 6).

All VFSSs were conducted in a purpose-built radiology suite on a Siemens Artis Fluoroscope (Siemens, German). All images were obtained at 30 frames per second in a lateral view. Radiation dose and time were recorded for each examination using the local hospital protocol. All VFSSs were conducted when the patient was medically fit for the procedure. A calibration ring (2.6 cm) was placed within the videofluoroscopic field of view to enable quantitative analysis to be completed. Patients were sat upright with support if required. A standardised VFSS protocol was used consisting of 1 ml, 3 ml, 20 ml and 100 ml continuous Level 0: Thin fluids (Varibar Barium Sulphate Contrast Agents 40% w/v, E-Z-EM Canada Inc.) and 5 ml paste (E-Z Paste). Studies were truncated for patient safety where necessary. All patients tolerated the 1 ml, 3 ml and 20 ml bolus presentations. Patients self-administered if able, otherwise they were provided with assistance. Standardised instructions were provided to each patient, “Put this in your mouth and hold it in your mouth until we say swallow”, “Swallow all in one go”, with the exception for the 100 ml continuous drinking, where they were asked to “swallow it all down as quickly as you are able”. On completion of the protocol, SLPs then had the opportunity to complete other trials as needed to answer the clinical question. On completion of the study, the images were converted in.avi files and uploaded into Swallowtail™ for analysis (Bell Dev Medical, LLC, Illinois, United States of America).

### Raters

Three analysis approaches were used in this study. Table 1 provides details of training, blinding and analysis method for each analysis approach. The traditional observational analysis groups were 9 treating SLPs and these SLPs were not provided with any instructions. They reported as per usual protocol at the hospital (Table 1). VFSS reports were retrospectively analysed to extract key information about PC, Hmax, PESmax and management recommendations.

**Table 1:** Description of analysis approaches

	Traditional observational analysis	Quantitative analysis	Binary subjective analysis
Number of raters	Nine SLPs	Two independent SLPs	11 independent SLPs
Videos rated	100 videos rated in agreement *by two treating SLPs* as per usual practice	100 videos rated by 1 SLP 20 random videos rated by a blinded SLP 20 random videos re-rated by one SLP 3 months later	100 videos rated by one SLP 40 videos rated by the other 10 SLPs—blinded
Training	Experienced SLPs (2–30 years) working with adult dysphagia and trained to use traditional observational VFSS analysis no training in quantitative measures	Experienced SLPs (> 5 years) working with adult dysphagia and trained to use the quantitative VFSS measures through video training, readings and mentorship	Experienced SLPs (> 2 years) working with adult dysphagia and trained to use traditional observational VFSS analysis no training in quantitative measures
Blinding	Full access to clinical records and the patient Blinded to quantitative analysis and binary subjective analysis	Provided with only patient gender and age. Blinded to traditional observational analysis and binary subjective analysis	Provided with patient gender and age only blinded to quantitative analysis and traditional observational analysis
Analysis	Reporting as per usual practice with ability to use frame-by-frame playback and unlimited time to review	Leonard and Kendall (1997) timing and displacement measures (Swallowtail)	Using frame-by-frame playback and unlimited time to review
Task	Judgement of PC, Hmax and PESmax retrospectively extracted from SLPs’ reports	Following measurement, quantitative raters rated PC using the PCR, Hmax, PESmax as within or outside 1SD of normative range	Requested to provide binary judgements of normal versus impaired for PC, Hmax, PESmax
Treatment	Treatment plan retrospectively extracted from SLPs’ reports	Asked to provide a treatment plan	No treatment plan requested

Quantitative analysis was based on the work of Leonard and Kendall [2] who published a standardised approach using quantitative, digital timing and displacement measures including our three metrics: pharyngeal constriction ratio (PCR), maximum hyoid displacement (Hmax) and maximum pharyngoesophageal segment opening (PESmax). PCR was calculated as a ratio between the measure of pharyngeal area at maximal constriction and the pharynx at rest. PESmax is a measure (cm) of the narrowest point of the PES between C4 and C6 during maximal opening during bolus transit. Hmax is a measure of distance (cm) between the hyoid at rest and at maximal excursion during the swallow. The age categories (> 65 vs < 65 years) and sex of each patient were provided to allow the SLPs to compare measures to norms. Two trained SLPs independently analysed the videos, with 20 randomly selected videos re-analysed 3 months later by the first rater to provide intra-rater reliability.

The binary observational analysis groups were 11 independent, blinded SLPs who were asked to provide binary judgements of ‘normal versus impaired’ for PC, Hmax, PESmax based on observational analysis. We provided no definition for ‘normal’ or ‘impaired’ and raters were encouraged to use their usual clinical judgement of what appears outside of normal function.

### Data Analysis

Data were collated in Microsoft Excel, and statistical analysis was completed using SPSS version 26 (SPSS, IL, USA). Inter-rater and intra-rater reliability of quantitative measures was calculated using intraclass co-efficient (ICC). Within analysis and between analysis approach agreement for the three binary metrics (PC, Hmax, PESmax) were analysed using Cohen’s kappa (*K*) to determine strength of agreement [23]. Agreement was qualified as 0.00, 0.10—virtually none; 0.11, 0.40—slight; 0.41, 0.60—fair; 0.61, 0.80—moderate; 0.81, 1.0—substantial [24]. Percentage agreements were also calculated to enable direct comparison to other similar studies where correlations were not calculated [21–23]. We decided on a cutoff greater than 70% agreement between binary subjective analysis as adequate agreement as there were very few (*n* = 5 or 4%) instances of 100% agreement.

Student t-tests were used to compare the frequency of recommendations of rehabilitation exercises, and percentage agreement was also used to compare the agreement of recommended rehabilitation exercises, between the quantitative analysis and the traditional observational approach. The Masako and effortful swallowing exercises were frequently recommended and directly reported due to their known ability to target pharyngeal constriction. Likewise, the Shaker head lift exercise and Mendelsohn Manoeuvre due to their known focus on pharyngoesophageal segment opening [25].

## Results

Inter- and intra-rater reliability for quantitative analysis was substantial across all measures (Table 2). Binary subjective analysis inter-rater reliability was fair across the three metrics (Table 2).

**Table 2:** Inter and intra-rater reliability for quantitative analysis and binary subjective analysis

	Inter-rater reliability			Intra-rater reliability		
	Inter-class co-efficient (ICC)	Confidence intervals	Strength of agreement	Inter-class co-efficient (ICC)	Confidence intervals	Strength of agreement
Quantitative analysis (*n* = 2) Raw scores						
OPT	0.773	0.36–0.93	Substantial	0.998	0.99–0.99	Substantial
HPT	0.866	0.58–0.96	Substantial	0.998	0.99–0.99	Substantial
TPT	0.889	0.64–0.97	Substantial	0.998	0.99–0.99	Substantial
PESdur	0.786	0.38–0.94	Substantial	0.987	0.93–0.99	Substantial
HDur	0.833	0.46–0.96	Substantial	0.767	0.44–0.99	Substantial
PCR	0.926	0.75–0.98	Substantial	0.887	0.70–0.96	Substantial
PESmax	0.934	0.78–0.98	Substantial	0.938	0.83–0.98	Substantial
Hmax	0.9	0.67–0.97	Substantial	0.874	0.67–0.96	Substantial
HLmax	0.876	0.61–0.97	Substantial	0.950	0.86–0.99	Substantial
Quantitative analysis (*n* = 2) Binary measure of within or outside 1SD of normative range						
	Kappa (*K*) score			Kappa (*K*) score		
PESmax	1.00	–	Substantial	1.00	–	Substantial
PCR	1.00	–	Substantial	1.00	–	Substantial
Hmax	1.00	–	Substantial	1.00	–	Substantial
Binary subjective analysis (*n* = 12) judgements of normal versus impaired						
PESmax	0.330	0.29–0.37	Fair	–	–	–
PCR	0.344	0.30–0.39	Fair	–	–	–
Hmax	0.356	0.31–0.40	Fair	–	–	–

*OPT* oropharyngeal transit time*, HPT* hypopharyngeal transit time, *TPT* total pharyngeal transit time, *PESdur* duration of pharyngoesophageal segment opening, *HDur* hyoid maximum duration, *PCR* pharyngeal constriction ratio, *PESmax* maximum opening of pharyngoesophageal segment, *Hmax* maximum hyoid displacement, *HLmax* maximum hyoid to larynx approximation

Across comparison groups, inter-rater agreement was slight to moderate. The highest percentage agreements achieved was for pharyngeal constriction and the lowest was for hyoid displacement (Table 3).

**Table 3:** Inter-rater agreement across analysis approaches

Metric	Comparisons	Kappa (*K*) score	Descriptor [21]	% Agreement
Pharyngeal constriction (PC)	Quantitative analysis vs Traditional observational analysis	0.210	Fair	62
	Quantitative analysis vs binary subjective analysis	0.457	Moderate	75
	Traditional observational analysis vs binary subjective analysis	0.279	Fair	73
PES maximum displacement (PESmax)	Quantitative analysis vs Traditional observational analysis	0.027	Slight	62
	Quantitative analysis vs binary subjective analysis	0.115	Slight	65
	Traditional observational analysis vs binary subjective analysis	0.416	Moderate	65
Hyoid maximum displacement (Hmax)	Quantitative analysis vs Traditional observational analysis	0.086	Slight	68
	Quantitative analysis vs binary subjective analysis	0.243	Fair	59
	Traditional observational analysis vs binary subjective analysis	0.317	Fair	63

### Diagnostic Accuracy

**Fig. 1:**
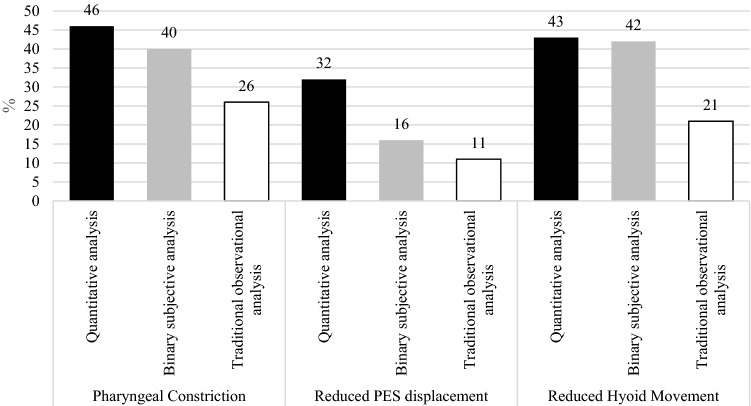
Percentage identification of impairment comparing analysis approaches

The most common of the three metrics identified as ‘impaired’ through quantitative analysis and traditional observational analysis was reduced pharyngeal constriction. In contrast, binary subjective analysis identified reduced hyoid movement most often. The least common was reduced pharyngoesophageal segment opening (PESmax) across all analysis approaches. Overall, the traditional observational analysis revealed the lowest percentage identification of impairments across all three analysis approaches (Fig. 1).

### Impact on Treatment Recommendations

Agreement was calculated for treatment recommendations between the quantitative and the traditional observational analysis. Across all four rehabilitation exercises included, there was only slight agreement achieved (Table 4).

**Table 4:** Inter-rater reliability for rehabilitation exercises

Rehabilitation exercises	Comparisons	Kappa score (*K*)	Descriptor [23]
Effortful swallow	Quantitative analysis vs traditional observational analysis	0.114	Slight
Masako	Quantitative analysis vs traditional observational analysis	0.189	Slight
Shaker head lift	Quantitative analysis vs traditional observational analysis	0.026	Slight
Mendelsohn	Quantitative analysis vs traditional observational analysis	0.019	Slight

**Fig. 2:**
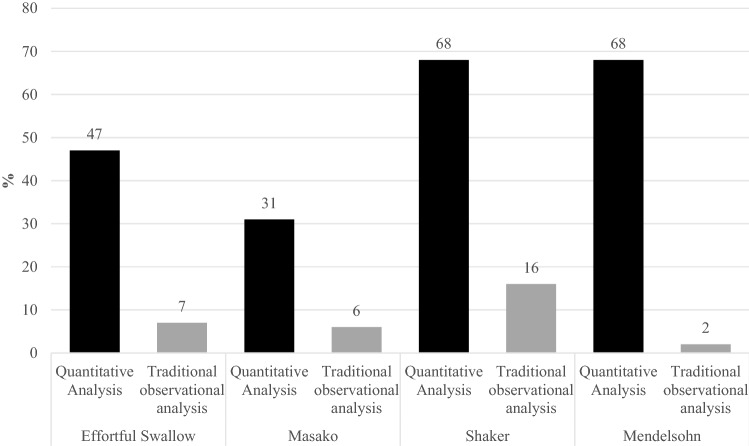
Percentage incidence of recommended rehabilitation exercises across analysis approaches

The traditional observational analysis clinicians prescribed rehabilitation exercises significantly less often than the quantitative analysis clinician (*t* = 4.84, *p* < 0.05) (Fig. 2). The highest percentage agreement was for Masako exercises at 73% and the lowest agreement was for Mendelsohn exercises at 35% agreement (Table 5).

**Table 5:** Percentage agreement for rehabilitation treatment between analysis approaches

Rehabilitation exercises	Quantitative analysis and binary subjective analysis	Quantitative analysis and traditional observational analysis
Effortful swallow	69	58
Masako	69	73
Shaker	54	39
Mendelsohn	54	35

## Discussion

VFSS is a commonly used diagnostic tool for assessing the biomechanics of swallowing and therefore critical for accurate diagnostics and targeted treatment planning [2]. VFSS also exposes patients to ionising radiation and thus optimising information that can be gathered from this study is imperative while complying with the As Low As Reasonably Achievable (ALARA) principle [26]. The results of this current study support previous findings of poor inter-rater reliability with traditional observational analysis across SLPs with various levels of experience [6, 7]. While it has been shown that analysis following group discussion improves inter-rater agreement [4, 27], this still does not address the accuracy of interpretation. This study demonstrated high inter- and intra-rater reliability when using quantitative measures to analyse VFSS, as well as increased accuracy of impairments through direct comparison with normative data. It also suggests that quantitative measurement leads to more treatment recommendations which are more consistent between SLPs, further demonstrating the potential significant positive impact of using quantitative measures.

It is acknowledged that VFSS can only give us a ‘snapshot in time’ and clinicians must consider this when making recommendations. However, quantitative measures also allow predictions of the probability that aspiration may occur for a given patient even if it is not witnessed during a single VFSS [2]. Prolonged pharyngeal transit times increase the risk of developing aspiration pneumonia, even if aspiration is not observed during VFSS [2, 19]. This likely relates to early entrance of the bolus into the pharynx prior to airway closure or longer dwell time of bolus with increased risk of post-deglutitive aspiration if residue is present. The pharyngeal constriction ratio is predictive of increased aspiration risk. Yip, Leonard and Belafsky [20] found statistically different PCRs between those who aspirated and those who did not, with increasing aspiration rates as the PCR deteriorated. Those with a PCR greater than 0.25 cm^2^ were three times more likely to aspirate than those with a normal PCR [2]. This predictive element to quantitative analysis enables the SLP to ascertain the impact of biomechanics on patient risk. Quantitative analysis in the current study produced a high level of agreement on PCR measures. In comparison, pharyngeal constriction had only moderate to fair agreement for both the traditional observational and binary subjective analysis. Consistent identification assists subsequent clinical decision-making.

### Clinical Impact of Quantitative Measures on Recommendations

Quantitative analysis, without knowledge of the patient history or exam findings, using only age, gender and quantitative VFSS measures, detected significantly more physiologic swallow changes and therefore generated more treatment recommendations compared to the traditional observational analysis where the full patient history and examination findings were known. There are many considerations for practicing SLPs when creating a treatment plan such as the physical or cognitive abilities of the patient. As the treating clinicians knew the patient’s history and previous examination findings, we acknowledge that this information may have contributed to altered treatment recommendations provided by them. However, to recommend a particular exercise or therapy, it is important to be able to first identify the impairment. The traditional observational approach identified significantly less impairment than the quantitative analysis, suggesting that clinicians using subjective analysis perhaps under-diagnose swallowing impairments and therefore, the full breadth of exercises that may have been physiologically appropriate for the patient were not considered. Both subjective analysis approaches were less accurate when compared to quantitative analysis results, regardless of whether the clinicians knew the patient or not. Interestingly, binary subjective analysis, where clinicians were only given limited patient information but were asked to rate in a binary fashion (normal vs impaired) for three chosen metrics performed better than the traditional observational raters who had full history and exam findings. This may be due to the binary selection having ‘primed’ or prompted these clinicians, however, from previous research we feel this is unlikely, as binary choices have been used in the past to increase reliability with little success [26, 27]. It seems more likely that the binary nature of the rating may have increased the likelihood of rating an impairment as present. In comparison, the treating clinicians may have omitted consideration of metrics in their self-directed reports.

Our findings also indicated that both groups often rated a parameter as normal if they were unsure. While this prevents clinicians over-diagnosing impairment, it also means that subtle impairments may not be identified without the aid of quantitative measurements. As a result, patients may not be offered appropriate treatment.

Recommendations for treatment should be based on accurate and reliable information while also taking into consideration risk factors and patient characteristics. The continued use of traditional observational analysis of VFSS alone may lead to under-diagnosing impairments, limited consideration of interventions and incorrect intervention recommendations. Quantitative measures provide both timing and displacement information and can be compared to normative data. This helps to benchmark the individual’s ability and inform treatment decisions. Timing disorders require a different management approach to motor deficits and quantitative measures potentially highlighting with increase accuracy possible areas of deficiency. Consideration of quantitative displacement and timing aspects of swallow gives a holistic picture and allows tailored interventions. The recommendations given by our quantitative analysis approach in this study were specific and because they are informed by findings on the VFSS, it gives confidence to the treating clinician in their management approach. It may therefore, avoid patients and clinicians putting time into a therapy programme that will not produce meaningful improvement in symptomatology. After a period of rehabilitation, repeating quantitative VFSS analysis will document responsiveness or identify remaining deficits. This may allow treatment choices to be adjusted if needed, based on physiological measures.

### Limitations

As we set out to analyse usual practice of treating SLPs, we did not prime them on what to report during their interpretations or provide them with education around interpretation. Radiological standards were sub-optimal at times, with missing views of vital structures and omission of the calibration ring, leading to the inability to utilise these studies in this research which the clinical team can learn from. Anterior–posterior (AP) view is not typically taken at this radiology site and the addition of AP view at other sites may change interpretation/agreement. Our data was extracted retrospectively and may not have identified other diagnostics that may have occurred. Future studies would benefit from a longitudinal approach to instituting a structured reporting of diagnostics and recommendations in treating clinicians.

Common language for description of VFSS findings and suggested management are in common parlance, however, individual-experienced SLPs may have adopted phrases that they are comfortable with that do not align with language sought in the VFSS reports. This may have led to detection bias which could be reduced in the future by utilising a comprehensive, yet finite list of terms that could be applied to VFSS reporting in future. The choice of a selected few strength-based treatment approaches was simplistic for illustration only and does not represent the full range of treatments available. This research would be supplemented by future studies on how patient outcomes, such as pneumonia rates, are impacted by implementation of quantitative measures.

## Conclusions

While VFSS is a commonly used tool to evaluate biomechanics of swallowing, this study suggests that incorporating quantitative measures into VFSS analysis identifies swallowing impairments with increased accuracy leading to an improved and more targeted set of treatment recommendations when compared to traditional observational analysis alone. Quantitative measures should be included alongside a traditional observational approach in clinical practice to ensure accurate and equitable identification of impairments, monitoring over time and reliability of recommendations. Since VFSSs are to remain a key tool in investigating swallowing difficulties, agreement in analysis is vital to allow research population comparisons and validate treatment approaches.
